# Fast quantitative reconstruction with focusing collimators for liver SPECT

**DOI:** 10.1186/s40658-018-0228-5

**Published:** 2018-12-04

**Authors:** Martijn M. A. Dietze, Sandra van der Velden, Marnix G. E. H. Lam, Max A. Viergever, Hugo W. A. M. de Jong

**Affiliations:** 1 Radiology and Nuclear Medicine, University Medical Center Utrecht, Utrecht University, P.O. Box 85500, 3508 Utrecht, GA Netherlands; 2 Image Sciences Institute, University Medical Center Utrecht, Utrecht University, P.O. Box 85500, 3508 Utrecht, GA Netherlands

**Keywords:** SPECT/CT, Reconstruction, Monte Carlo, Quantification, Radioembolization

## Abstract

**Background:**

Generation of a SPECT scan during procedure may aid in the optimization of treatments as liver radioembolization by offering image-guided dosimetry. This, however, requires both shortened acquisition times and fast quantitative reconstruction. Focusing collimators increase sensitivity and thus may speed up imaging. Monte Carlo-based iterative reconstruction has shown to provide quantitative results for parallel hole collimators but may be slow. The purpose of this work is to develop fast Monte Carlo-based reconstruction for focusing collimators and to evaluate the impact of reconstruction and collimator choice on quantitative accuracy of liver dosimetry by means of simulations.

**Results:**

The developed fast Monte Carlo simulator was found to accurately generate projections compared to a full Monte Carlo simulation, providing projections in several seconds instead of several days. Monte Carlo-based scatter correction was superior to other scatter correction methods in describing recovered activity and reached similar noise levels as dual-energy window scatter correction. Although truncation artifacts were present in the cone beam collimator (50 cm), the region inside the field of view (FOV) could be reconstructed without loss of accuracy. Provided the object to image is inside the FOV, the focusing collimator with 50 cm focal distance could retrieve the same noise levels as a parallel hole collimator in 68% of the total scanning time, the multifocal collimator in 73% of the time, and the 100-cm focal distance collimator in 84% of the time.

**Conclusion:**

Focusing collimators combined with Monte Carlo-based reconstruction have the ability to enable quantitative imaging of the FOV in a significantly shorter timeframe. The proposed approach to the forward projector will additionally make it possible to reconstruct within minutes. These are crucial steps in moving toward real-time dosimetry during interventions.

## Background

Liver malignancies are increasingly treated with intra-arterial microsphere radioembolization [1, 2]. In this procedure, beta-emitting radioactive isotopes as yttrium-90 (^90^Y) (encapsulated by either glass (TheraSphere; MDS Nordion, Ontario, Canada) or resin (SIR-Spheres; Sirtex Medical, Sydney, Australia)) and holmium-166 (^166^Ho) (QuiremSpheres; Quirem, Deventer, The Netherlands) are injected into the hepatic artery. The microspheres distribute according to the vascularity, ideally occluding small tumor capillaries in which they deliver a high dose to the tumor while sparing the healthy parenchyma.

Single-photon emission computed tomography (SPECT) of technetium-99m macroaggregated albumin (^99m^Tc-MAA) is normally performed before treatment to study the sphere deposition in the liver and to ascertain minimal dose to healthy liver parenchyma and lungs. Ideally, this scout scan would be performed shortly before the treatment to minimize changes in pathology and catheter position. In addition, if the scout scan proves affirmative, it can be directly followed by the treatment in a single procedure. Such image-guided procedures are, however, not commonly practiced because SPECT/CT is not available in the intervention room, and the combined time of transporting the patient to the scanning room and performing a SPECT scan is too long to hold the intervention room in most hospitals [3]. We envision a gamma camera in the intervention room to eliminate the need for patient transportation. Additionally, if such a device were to acquire accurate SPECT images in a matter of minutes, real-time image-guided radioembolization procedures would become feasible.

**Fig. 1:**
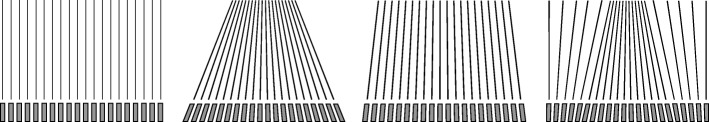
Illustration of the field of view (FOV) of the discussed collimators. From left to right: parallel hole collimator, cone beam collimator (50 cm), cone beam collimator (100 cm), and multifocal collimator

To reduce the SPECT scanning times, we propose to use focusing collimators to increase sensitivity. Historically, this has been done in brain SPECT using a cone beam collimator [4, 5], inasmuch as the object to reconstruct is smaller than the size of the gamma camera. More recently, a multifocal collimator (e.g., SmartZOOM on the IQ-SPECT) for cardiac SPECT has been introduced in clinical practice [6, 7], with the aim of improving sensitivity in the area of interest, while capturing the unfocused photons to reduce truncation artifacts (see Fig. 1). Such collimators could function in radioembolization SPECT similarly, since the volume of dosimetric interest is often located in a predetermined region (e.g., retrieved from a CT or MRI scan).

For image-guided procedures to be successful, not only the acquisition stage should be fast, but quantitative reconstruction should also be performed in a matter of minutes. To ensure that image quality is preserved while reducing reconstruction time, sophisticated reconstruction algorithms can be used. This study will make use of Monte Carlo-based iterative reconstruction, which has previously been shown to improve quantitative results for several isotopes and parallel hole collimators [8–10]. Focusing collimators for these reconstructions introduce new characteristics, such as a shift-variant point spread function (PSF) and potentially severe truncation artifacts. The purpose of this work is to evaluate to what extent the combination of Monte Carlo-based iterative reconstructions and focusing collimators can realize the need for fast and quantitatively accurate liver SPECT imaging in the intervention room, by means of a simulation study.

## Methods

### Simulated collimators

Four specific configurations were considered for the comparison of focusing collimators: the regular parallel hole collimator, cone beam collimators with focal lengths of 50 cm and 100 cm, and a multifocal collimator [11] (Fig. 1). These collimators were chosen as they are often already available at hospitals (or can be readily made) for the previously mentioned procedures of brain and cardiac imaging.

To be able to make accurate comparisons, the collimator parameters were standardized. All collimators were equipped with LEHR specifications (24.05 mm hole length, 1.11 mm hole diameter, 0.16 mm septal thickness), as is customary for ^99m^Tc imaging [12], and covered a camera crystal surface of 53.3 × 38.7 cm^2^ (Siemens Symbia T characteristics [12]). The multifocal collimator was equipped with a focal distance of 50 cm and a focusing area covering half of the total surface: 26.7 × 19.4 cm^2^.

**Fig. 2:**
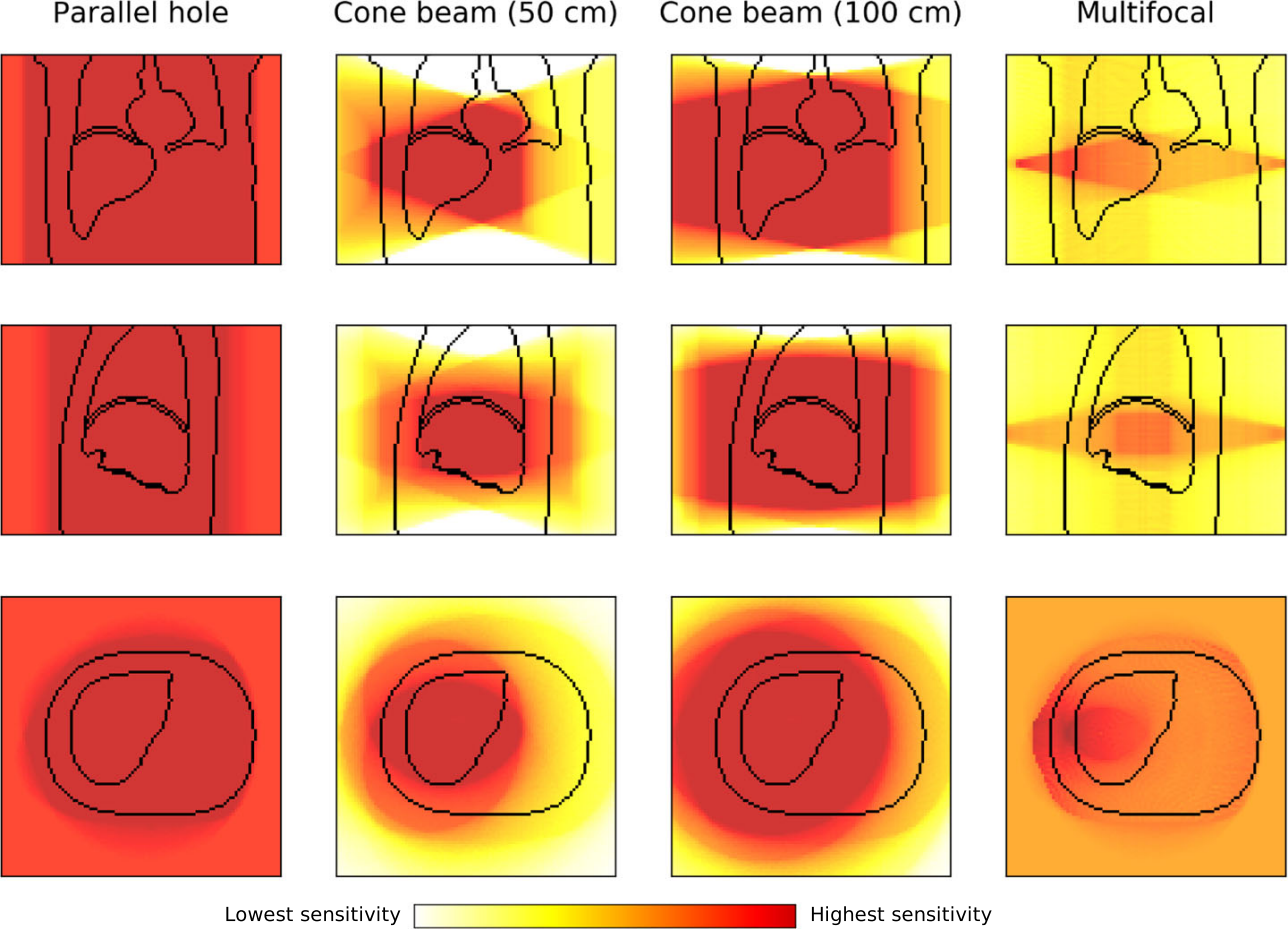
From top to bottom are the coronal, sagittal, and transverse planes of the XCAT phantom (drawn are the body, liver, and lung contours) for the simulated collimators. The color gives an indication of the geometric sensitivity of each collimator. The detector surface follows a closed (non-circular) contour of the patient while rotating around the center of the liver

To illustrate the effect of the varying field-of-views of the different collimators with respect to liver coverage, the XCAT phantom [13] is shown together with the four collimators rotating at a distance of 1.0 cm from the body in Fig. 2. The parallel hole collimator has no spatial dependence and has thus equal information over the entire liver. The cone beam collimator (50 cm) has complete information of only part of the liver, while the cone beam collimator (100 cm) is able to encapsulate the entire organ. The multifocal collimator images show part of the liver with increased sensitivity and the remainder with decreased sensitivity. Notably different from the usual cardiac imaging is that not one sharply defined sphere with increased sensitivity is imaged, but, since the liver lies more off-center in the body than the heart, this volume is smeared out over a larger volume.

The cone beam collimators may be prone to truncation artifacts. When a large fraction of activity is present outside the area that is imaged in most projections, this could result in incorrect quantification. The multifocal collimator has a FOV equal to that of a parallel hole collimator and hence does not have this problem. However, this geometry results in a decrease in sensitivity in the background, which might reduce the advantage of the focusing geometry. The magnitude of these effects was investigated using a Monte Carlo simulation study.

### Point spread function modeling

**Fig. 3:**
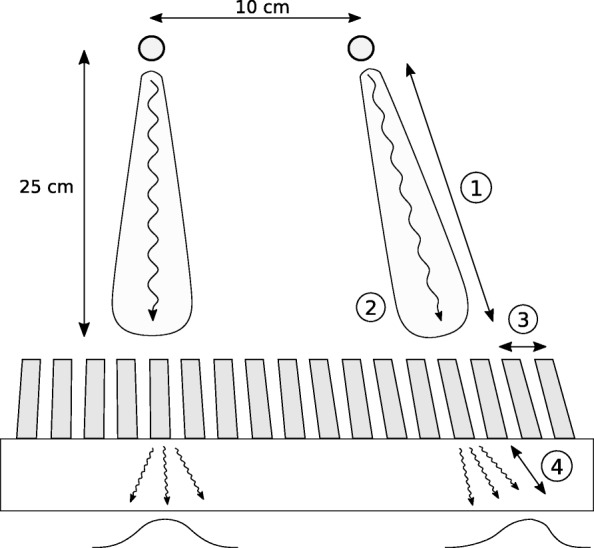
Differences between a point source located in the middle of a focusing collimator versus one located toward the side. The effects corresponding to the numbers can be found in the text. It can be expected that the PSF of a point source located in the origin has a different shape than a point source toward the edge; the severity of this effect was studied in a simulation with point sources as located in the figure

The Utrecht Monte Carlo System (UMCS) is a Monte Carlo-based iterative SPECT reconstruction package. UMCS is able to simulate photon interactions inside the body in a fast manner, so that implementation in the clinic is feasible. The forward projector has previously been validated for ^99m^Tc, ^90^Y, and ^166^Ho; a complete description can be found elsewhere [8–10]. UMCS applies convolution-based forced detection (CFD), which projects a point spread function (PSF) from every source or scatter position to the camera. To implement CFD for focusing collimators, the object transformation was performed using rotating and warping in a single interpolation step [14]. Additionally, several characteristics of focusing collimators differ from those of regular parallel-hole collimators and affect the shape and magnitude of the PSF (see Fig. 3):

1. The distance of a point source to the collimator surface increases with spatial distance from the middle of the collimator, broadening the PSF.
2. There is a change in coordinate system as the collimator captures photons over a sphere, while the detector is flat. This introduces a Jacobian determinant of 1/cos^2^(*θ)* (with *θ* being the angle of the irradiated collimator hole) [14] and thus induces spatial dependence on gamma camera sensitivity.
3. Hole diameter and septa thickness increase with distance from the origin, changing the number of photons that interact with the collimator.
4. The attenuation of photons in the crystal increases with distance from the origin. Since photons enter the crystal under an angle, this makes the shape of the PSF slightly asymmetric.

To study the severity of the above effects on the shape of the PSF, a simulation study was performed on the cone beam (50 cm) collimator. The collimator configuration was modeled in GATE [15] with one point source positioned at the center of the collimator and one at the edge of the collimator (with a spatial shift of 10 cm), both at a distance of 25 cm from the gamma camera (see Fig. 3). This is a region in which the greatest deviation between PSFs is expected, because the offset point source is detected close to the edge of the gamma camera. The differences in magnitude and shape of the retrieved PSFs were measured for comparison.

### Acceleration of shift-variant PSF modeling

As mentioned, the shift variance of the PSF will be greatest for regions toward the side with a large distance from the detector. For the other regions, the effect will likely be not significant in comparison to the intrinsic detector resolution. We propose that it is sufficient to perform only a single convolution per focusing/diverging region to accurately describe the detector physics, thus only accounting for the Jacobian determinant. The shift variance of the PSF was thus not included in the reconstruction. This may slightly reduce the accuracy, but will greatly improve the reconstruction speed.

Thus, for the cone beam collimators, one convolution per step in height is required over the *x/y*-plane to model the detector physics; for the multifocal collimator, four regions are defined: one with focusing in both directions, one with focusing in *x* and divergence in *y*, one with divergence in *x* and focusing *y*, and finally one with divergence in both directions. The regions with combined focusing and divergence have an elliptical shape and thus no rotational averaging was performed. The border where the different regions meet was averaged over a width of two pixels.

### Projector evaluation

To evaluate the performance of the proposed fast forward projector, projections of an image quality phantom (IEC NEMA 2007; PTW, Freiburg, Germany) were compared against full Monte Carlo simulation in GATE for all collimator configurations. The spheres of the digital NEMA phantom were configured with a 10:1 background ratio of ^99m^Tc and photons were generated until the smallest sphere was visually distinguishable from the background. The profiles of the spheres in the projections were compared to verify whether the PSF was indeed correctly modeled and whether the sensitivity of the system was accurate. The speed of both simulators was compared to find the acceleration factor of the proposed fast Monte Carlo projector over the full one.

### Digital phantom study

To evaluate the performance of the mentioned collimators for quantitative liver SPECT, reconstructions of the XCAT liver and lung were made. A tumor (radius of 20 mm; positioned in the converging region of all collimators) was inserted into the liver at a 5:1 uptake ratio. The total activity in the phantom was 100 MBq. The lung was filled uniformly with a lung shunt fraction (LSF) of 5%. To speed up the simulations, the fast forward projector of UMCS was used to create projections, on which Poisson noise was added to emulate a clinical setting.

The obtained projections were subsequently reconstructed with attenuation and scatter correction and resolution recovery (PSF modeling) in both forward and backward projections, with eight subsets for ten iterations. The photon physics were simulated based on the phantom density map in the forward projection. Ten noise realizations were performed to retrieve an indication of the stability of the reconstructions. The phantoms were configured in a 128 × 128 × 90 grid, with 4.7 mm isotropic voxels. The energy window was set at 15% around the 140 keV photopeak.

The effect of truncation on the reconstruction was evaluated by reconstructing the liver phantom with and without physical camera size; the latter assumes an infinitely large detector surface. The reconstructed activity in the tumor was measured to study whether truncation affects the quantitative results inside the FOV.

For comparison of the Monte Carlo-based scatter correction, reconstructions were also performed with a dual-energy window scatter correction [16] and without scatter correction. In the case of dual-energy window scatter correction, projections of the scatter window (20% around 110 keV) were additionally simulated—again with Poisson noise added. These scatter projections were smoothed by a Gaussian filter of two pixels, added to the reconstruction loop and weighted with a *k* factor for the scaling of the difference of scatter in the photopeak window and the scatter window, which was evaluated at the clinically used value of *k* = 0.5 and an optimal value. This optimal *k*-value was first based on the ratio of the scatter in the photopeak and the scatter energy window and was then further tuned by additionally simulating more *k*-values both above and below this first estimate. The *k*-value resulting in the lowest mean squared error with the phantom over the entire liver was used as the optimal value. The recovery profiles of the different scatter corrections methods were measured to study their effect on tumor contrast.

Subsequently, the noise properties of the collimators and scatter correction methods were studied in more detail. To evaluate whether they are able to reduce scanning times, reconstructions were performed for several view durations (20 s—as is clinical protocol, 17.5 s, 15 s, 12.5 s, 10 s, 7.5 s, and 5 s). A tumor VOI was created by selecting the tumor region and eroding it with one pixel to reduce partial volume effects. As the total reconstruction quality is of primary interest, the spatial noise dependence was not studied. Therefore, the background liver VOI, for which no truncation artifacts were present, was created by selecting the hot region for the cone beam (50 cm) collimator from Fig. 2 and subtracting the tumor VOI. The lung VOI was created similarly, except that no tumor VOI had to be subtracted.

## Results

### Point spread function modeling

**Fig. 4:**
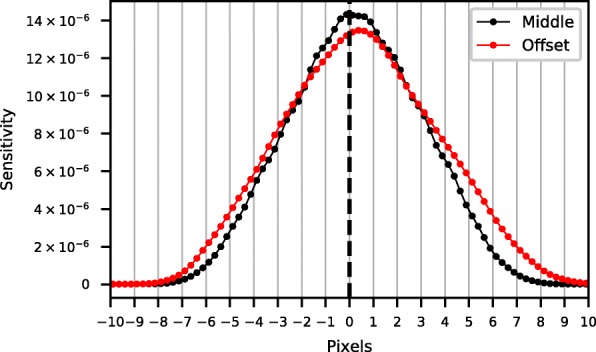
Difference in point spread function for a point source located in the origin at *z* = 25 cm and one with an offset of *dx* = 10 cm. These PSFs were corrected for the 1/cos^2^ (*θ*) sensitivity factor

The profiles of the simulated point sources in the origin and at the edge of the cone beam collimator (50 cm), blurred with the intrinsic gamma camera resolution of 3.8 mm full-width at half-maximum (FWHM) [12], are shown in Fig. 4. It can be seen that the difference between the means of the distributions is less than one pixel (4.7 mm). This means that, other than the 1/cos^2^ (*θ*) Jacobean factor introduced by the coordinate change, the shape and sensitivity of the PSF only changed slightly.

### Projector evaluation

**Fig. 5:**
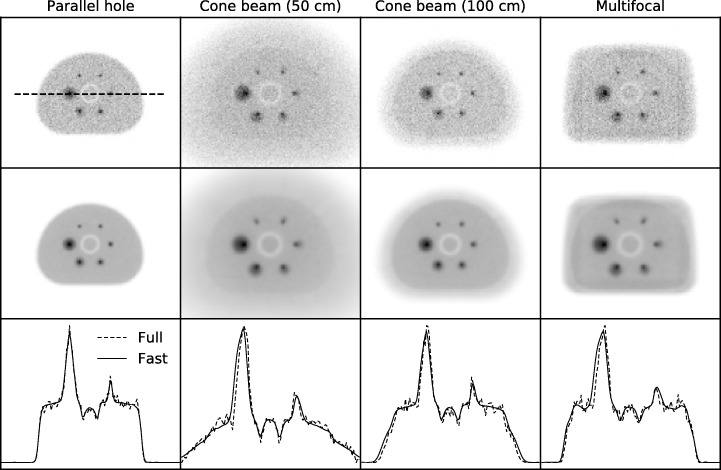
Projections of the NEMA phantom made with the GATE Monte Carlo projector (top row) and with the fast Monte Carlo projector (middle row) for the parallel hole, cone beam (50 cm), cone beam (100 cm), and multifocal collimators. Profiles along the middle spheres are shown in the bottom row. The GATE Monte Carlo projections are generated with 2.6, 4.1, 2.7, and 2.6 billion photons, respectively

The projections of the NEMA phantom simulated with the fast Monte Carlo simulator (UMCS) and the full Monte Carlo simulator (GATE) are shown in Fig. 5. The profiles over the middle spheres are visualized and agree well with each other. Hence, the proposal of a single convolution per focusing region is sufficient and UMCS can be used as a quantitative forward projector for focusing collimators.

The fast forward projector was able to generate a projection in approximately 2 s, while the full Monte Carlo projection took 4 days (single-threaded) to obtain images with a quality as in Fig. 5 on a regular desktop PC. The total reconstruction time for the ten iterations with the mentioned matrix configuration using the fast forward projector was approximately 1 h.

### Digital phantom study

**Fig. 6:**
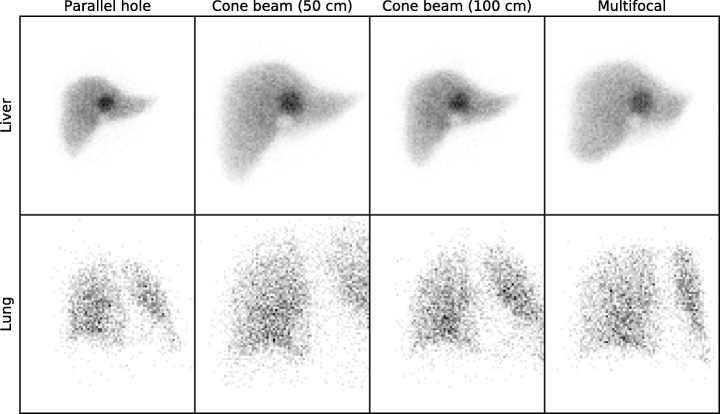
Projections of the XCAT liver and lung made with the fast Monte Carlo projector, with Poisson noise added to emulate a detector measurement of 20 s. The number of counts in the liver projections is 56 k, 104 k, 75 k, and 90 k, respectively. For the lung, these are 3458, 6376, 5406, and 5144, respectively

Projections of the XCAT lung and liver (with added Poisson noise) can be found in Fig. 6. The number of counts for the focusing collimators was higher than for the parallel hole geometry. Since the resolution of all collimators is equal, more accurate reconstruction results should therefore be possible with the focusing collimators.

**Fig. 7:**
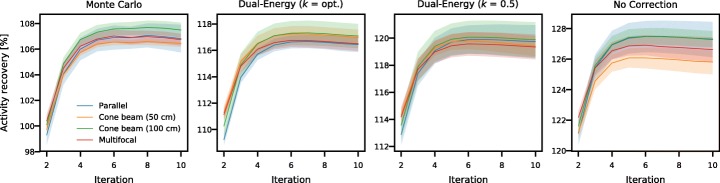
Activity recovery in the tumor, collected per scatter correction method. The shaded bars indicate the standard deviation retrieved from the ten noise realizations

The activity recovery (activity of the reconstruction divided by activity of the phantom) in the tumor with the various collimators is presented in Fig. 7. Convergence rate is approximately the same for all collimators. However, absolute activity recovery values differ for the various methods of scatter correction: approximately 106% is retrieved for the Monte Carlo-based method, 116% for the dual-energy window method with optimal *k*, 119% with clinical *k* = 0.5, and 127% for no scatter correction applied. As the activity recovery was found to be converged after ten iterations, this setting was used in the remaining analysis.

**Fig. 8:**
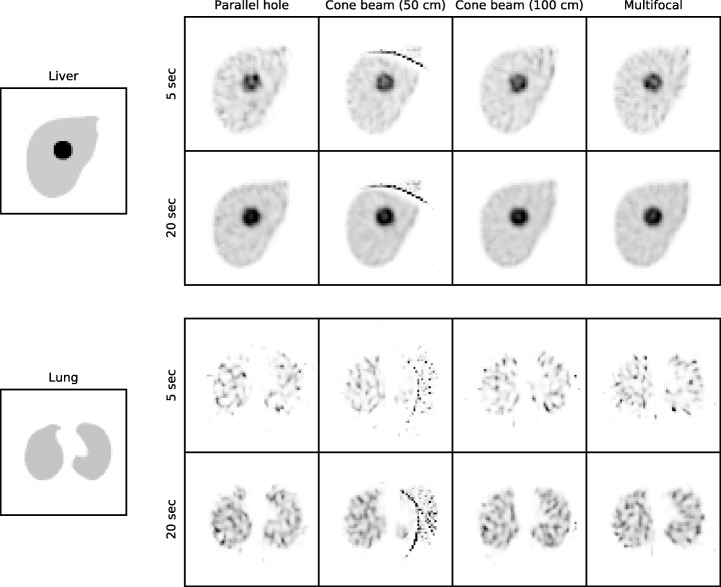
Reconstructions of the XCAT liver and lung for all collimators for 5 and 20 s per view reconstructed with Monte Carlo-based scatter correction

Reconstructions of the digital phantoms are displayed in Fig. 8, shown separately for reconstructions of 5 and 20 s per view. It can be observed that the background noise was reduced in the reconstructions of the focusing collimators. The lung reconstructions possessed higher noise levels than those of the liver, due to their lower activity. It is furthermore evident how truncation influenced the results, inasmuch as in the cone beam (50 cm) reconstruction streak artifacts appeared toward the edge of the phantoms. The objects within the FOV, however, seem to be reconstructed correctly for all collimators.

### Truncation

**Fig. 9:**
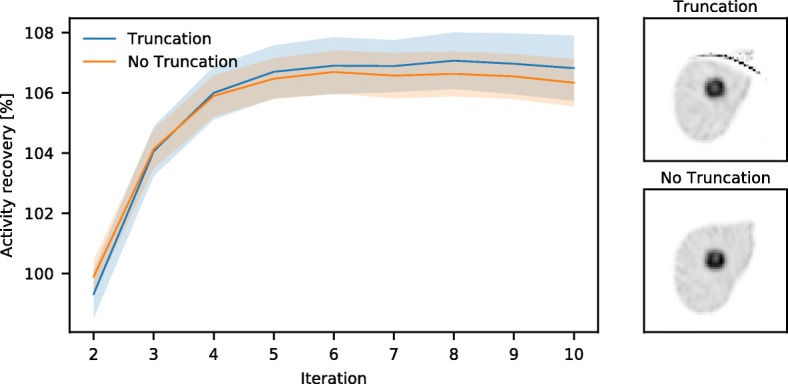
Activity recovery of reconstructions with and without truncation, for the cone beam (50 cm) collimator reconstructed with Monte Carlo-based scatter correction

To study the effect of truncation, the activity recovery in the tumor of the liver reconstructions with and without truncation was compared, for the cone beam (50 cm) collimator; see Fig. 9. The reconstructions with truncation converge toward the same value as those without truncation. Hence, truncation artifacts do not significantly influence the quantitative accuracy inside the FOV for this configuration. The volume where no artifacts were present (i.e., the volume seen by more than half of the total number of projections) was found to be 86% of the liver and 51% of the lung total volume.

### Scatter correction

**Fig. 10:**
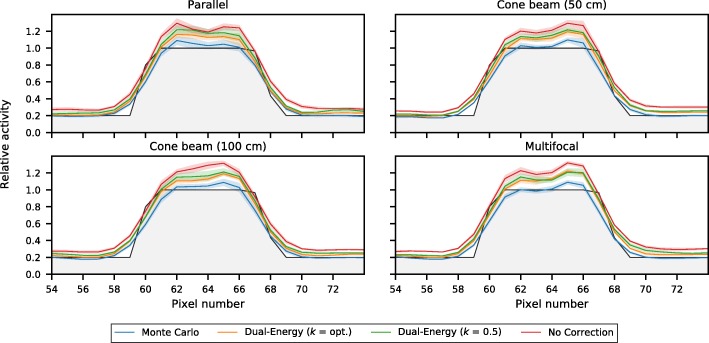
Activity recovery profiles, normalized to the maximum activity, of the investigated scatter correction techniques for the several collimators. In black is the phantom profile

To study the effect of scatter correction, activity recovery profiles of the various scatter correction techniques were generated; these are shown in Fig. 10. It is evident that reconstruction without scatter correction leads to an overestimation of activity over the entire profile. Dual-energy window scatter correction was able to trace the contours better, since the scattered photons are added to the reconstruction loop. Dual-energy window scatter correction with optimal *k* factor (found to be *k* ~ 0.72 for all collimators) performed better than with clinical *k* = 0.5 for this phantom, especially in the background region. Both options however have difficulty with correct quantification of the tumor. Monte Carlo-based scatter correction was able to trace the contours most accurately, both in the background and around the tumor.

**Fig. 11:**
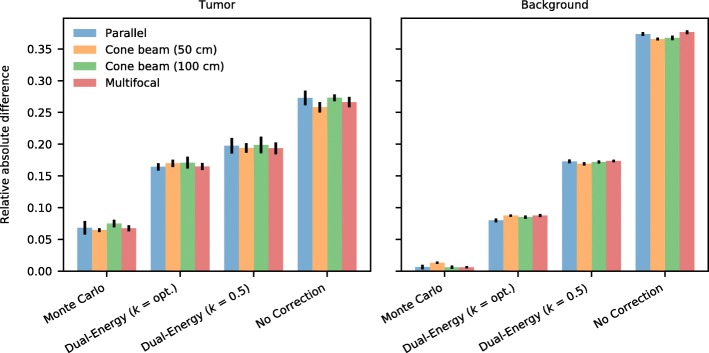
The relative absolute difference, normalized to the mean activity, of the reconstructions with the phantom (ground truth), shown separately for the tumor and the background region, for the four collimators

These results were further detailed by calculating for each of the collimators the absolute difference with the ground truth (phantom), shown in Fig. 11 separately for the tumor and the background VOI of the liver phantom. It can again be observed that Monte Carlo-based scatter correction outperforms the other options.

### Scan time reduction

**Fig. 12:**
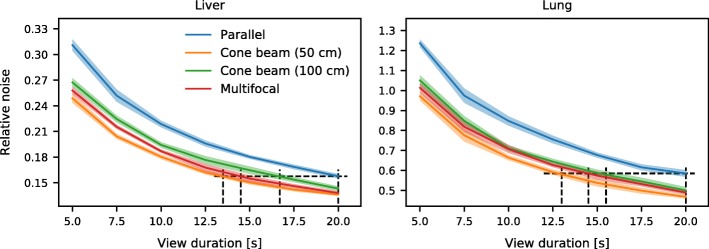
Noise levels, normalized to the mean activity, for the four collimators obtained by reconstruction with Monte Carlo-based scatter correction. The dotted framework shows the time needed for the cone beam and multifocal collimators to achieve the same image quality as the parallel hole collimator at the clinical setting of 20 s per view

To study the effect of scanning times on the reconstructions, projections of the digital phantoms were simulated for several view durations. It can be seen in Fig. 12 that the focusing collimators yielded a reduced noise level in comparison to the parallel hole collimator. The trends in the liver and the lung phantom were comparable.

The total possible time gain of the focusing collimators can be found by calculating the points on the curves where a similar noise level is achieved as with the parallel hole collimator at 20 s per view (clinical setting). This shows that comparable quality can be achieved in approximately 13.5 s per view for the cone beam (50 cm) collimator (thus 68% of the total scanning time), 14.5 s per view (73% of the scanning time) for the multifocal collimator, and 16.7 s per view (84% of the scanning time) for cone beam (100 cm) collimator.

**Fig. 13:**
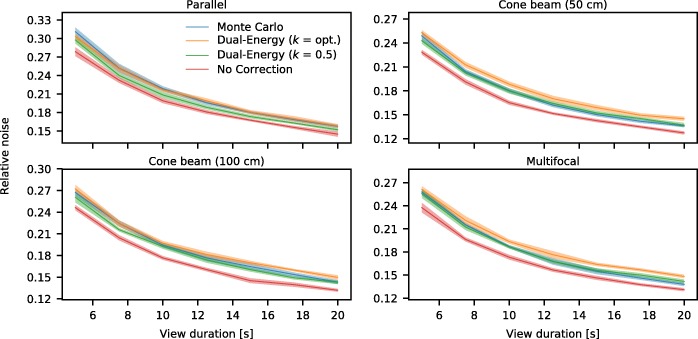
Noise levels, normalized to the mean activity, for the scatter correction methods and for the reconstruction without scatter correction, for the four considered collimators

Similarly to the noise properties of the focusing collimators with respect to the parallel hole collimator, the effects in scatter correction method can be evaluated. These results are presented in Fig. 13 for all considered collimators. No scatter correction has the lowest level, as no corrections are performed for this option. Monte Carlo-based and dual-energy window scatter correction can be found to achieve comparable noise levels.

## Discussion

This study investigated to what extent quantitative liver SPECT imaging could be accelerated by combining focusing collimators with Monte Carlo-based reconstruction. We showed that focusing collimators are able to reduce scanning times in comparison to the clinical procedure—provided that the object to image is inside the FOV. The proposed approach to the forward projector allows fast reconstruction and gives quantitative results. This is an important step in realizing real-time image-guided radioembolization procedures.

No definitive conclusion was reached in this work as to which collimator is best suited for liver SPECT, since this strongly depends on liver size and tumor locations. We therefore consider this an exploratory study in which the implementation of fast reconstruction of focusing collimators is discussed and the individual effects of truncation and scatter are shown. In the case of radioembolization, it is desired to have homogeneous image quality over the entire liver. This would favor the cone beam (100 cm) or multifocal collimator. However, in the case of a solitary tumor, the cone beam (50 cm) collimator would be more beneficial.

The SPECT/CT scans required for radioembolization do not necessarily have to be performed with 20 s per view. It may be that reconstructions gathered with a shorter time per view are also of sufficient quality for clinical use. However, also for these faster scans, there will be a benefit in using focusing collimators. For a 10 min scan, e.g., one could move toward ~ 7 min scanning time. With the aim of interventional scanning as described in this work, the gain of a few minutes in scanning time could be crucial to facilitate implementation in the clinic.

Other than the localization of activity distributions, one clinical use of the ^99m^Tc scout dose is the determination of the LSF. As discussed, the FOV of the focusing collimators is smaller than that of the parallel hole collimator, which therefore potentially requires multiple separate acquisitions to also cover the lung region. However, as the LSF is determined over two large regions, it is much less sensitive to low count rates. It is envisioned that one or two acquisitions can thus be quickly (likely < 1 min) performed to calculate the LSF. This LSF procedure will thus likely not be limiting for the real-time image-guided procedures.

This work has studied ^99m^Tc for use as scout dose to facilitate fast implementation in the clinic. Promising research is however ongoing on the use of ^166^Ho [17, 18] as a scout dose for the corresponding liver radioembolization, so that scout and treatment particle are equal. This will be investigated for ^90^Y similarly. For these isotopes, bremsstrahlung will be the most important measure and scatter modeling will have an even larger impact than for ^99m^Tc imaging. It will be straightforward to extend this simulation study to the above-mentioned isotopes by generation of new point spread functions.

Focusing collimators are not the only option for scan time reduction. Other options are, for instance, gating of the respiratory signal [19], so that motion artifacts are suppressed, and optimization of the time spent per view [20], so that more counts are collected in the projections that carry most information. Ideally, all these options would be combined in a single system.

SPECT reconstruction with Monte Carlo-based scatter correction comprising ten iterations with clinical parameter settings with our implementation currently takes approximately 1 h on a single-threaded regular desktop. Obviously, this is too long for inclusion into an image-guided procedure. The most time-consuming step in this reconstruction is the rotating and warping transform, due to the many memory look-up operations required. If this step is optimized and implemented on a GPU-based workstation, we expect that reconstruction in the order of minutes is achievable.

Commercial reconstruction software is generally already able to retrieve reconstructions within a few minutes, thus too facilitating real-time image-guided procedures. These packages however usually employ dual-energy window scatter correction, of which it was shown that quantification fails for smaller distributions. For such distributions, the more extensive Monte Carlo-based scatter correction would be better suited to accurately describe the true activity.

In convolution-based forced detection, many options to model the PSF have been used. Du et al. [21] warped a model for parallel hole geometry into the focusing geometry, Tsui and Gullberg [22] and Van Roosmalen and Goorden [23] employed analytic functions for the representation, and Chun et al. [24] took the response from the detector itself and generalized this, which requires measurements for all distances, isotopes, and collimators. All implementations have their own strengths and weaknesses and the best option will depend on the individual requirements. In the case of ^99m^Tc modeling, collimator interactions will be minimal and hence all options can be expected to achieve similar results. However, when modeling higher energy photons (e.g., ^90^Y, ^166^Ho), collimator interactions will likely become a major factor. In this setting Monte Carlo-based PSFs are expected to most accurately describe the detector physics.

To achieve the largest FOV in the liver, one option would be to increase the gamma camera size. However, as the discussed collimators are symmetric, the gamma camera would at some point collide with the shoulder of the patient. These problems have also been encountered in brain SPECT, where the gamma camera cannot be positioned as close to the skull as preferred, again since the shoulder gets in the way. To solve this problem, a half-cone beam collimator was proposed [25]. Such a tilted collimator could be of use for liver SPECT as well.

The reconstructions performed in this work were made from projections using the same forward projector. This means that the reconstructions will iterate toward the original phantom and are not the same as detector projections [26]. Previously, however, it has been shown that measured detector data match the simulated projections closely [27, 28], and hence, we assume that these simulated projections provide a good starting point for the investigation of focusing collimators. Future work will focus on the reconstruction of detector measurements.

Comparison of the collimators using detector measurements will however be challenging, as there exist several parameters that influence the system sensitivity. For example, small production errors in the collimator, differences in photomultiplier tube gains, and varying crystal efficiency can all influence the observed noise levels. Furthermore, the used phantom must be configured exactly the same during every measurement. In this study, it was therefore decided to only focus on simulations, as this makes it able to only study the effect of collimator choice.

As mentioned in the introduction, a gamma camera in the intervention room would be ideal for image-guided liver SPECT. The patient then does not have to be transferred to the scanning room, which saves time and reduces patient discomfort. Significant effort has recently gone into the development of mobile gamma cameras [29, 30]. If such a device were included in the intervention room, the SPECT scan can instantly be made after the scout dose in a short time frame, which would substantially expedite the procedure.

## Conclusions

A fast quantitative Monte Carlo reconstructor for focusing collimators was developed, making reconstruction within minutes feasible. The use of focusing collimators for liver SPECT decreased scanning times. Monte Carlo-based scatter correction traced the tumor activity profiles most accurately. Both fast acquisition and fast quantitative reconstruction are crucial steps in moving toward real-time dosimetry during interventions.

## Abbreviations

^166^Ho: Holmium-166
^90^Y: Yttrium-90
^99m^Tc-MAA: Technetium-99m macroaggregated albumin
CT: Computed tomography
FOV: Field of view
FWHM: Full width at half maximum
LSF: Lung shunt fraction
PSF: Point spread function
SPECT: Single-photon emission computed tomography
VOI: Volume of interest
